# Statistical analysis plan for the EuroHYP-1 trial: European multicentre, randomised, phase III clinical trial of the therapeutic hypothermia plus best medical treatment versus best medical treatment alone for acute ischaemic stroke

**DOI:** 10.1186/s13063-017-2302-z

**Published:** 2017-11-29

**Authors:** Per Winkel, Philip M. Bath, Christian Gluud, Jane Lindschou, H. Bart van der Worp, Malcolm R. Macleod, Istvan Szabo, Isabelle Durand-Zaleski, Stefan Schwab, for the EuroHYP-1 trial investigators

**Affiliations:** 1 The Copenhagen Trial Unit, Centre for Clinical Intervention Research, Blegdamsvej 9, Copenhagen, Denmark; 2 Stroke Trials Unit, Division of Clinical Neuroscience, University of Nottingham, Nottingham, NG5 1PB UK; 3 Department of Neurology and Neurosurgery, Brain Center Rudolf Magnus, University Medical Center Utrecht, Heidelberglaan 100, 3584 CX Utrecht, The Netherlands; 4 Division of Clinical Neurosciences, University of Edinburgh, FU204, Chancellor’s Building, Royal Infirmary of Edinburgh, Little France Crescent, Edinburgh, EH16 4SA UK; 5 European Stroke Research Network for Hypothermia, Square de Meeus 38/40, 1000 Brussels, Belgium; 6 ECEVE, UMR 1123, URCEcoIle de France Hôpital de l’Hotel Dieu, 1 Place du parvis de Notre Dame, F 75004 Paris, France; 7 Neurologische Klinik, Universitætsklinikum Erlangen, Schwabachanlage 6, 91054 Erlangen, Germany

**Keywords:** Acute ischaemic stroke, Randomised clinical trial, Modified Ranking scale, Quality of life, Cooling, Cost-effectiveness

## Abstract

**Background:**

Cooling may reduce infarct size and improve neurological outcomes in patients with ischaemic stroke. In phase II trials, cooling awake patients with ischaemic stroke has been shown to be feasible and safe, but the effects in functional outcomes has not yet been investigated in an adequately sized randomised clinical trial.

**Methods/design:**

The EuroHYP-1 trial is a multinational, randomised, superiority phase III clinical trial with masked outcome assessment testing the benefits and harms of therapeutic cooling in awake adult patients with acute ischaemic stroke. The outcomes dealt with here include the *primary outcome* the Rankin score (mRS) at day 91 +/-14 days after randomisation. The *secondary and exploratory outcomes* at day 91 +/-14 days unless otherwise stated encompassing: (1) death or dependency, defined as mRS score > 2; (2) death; (3) National Institutes of Health Stroke Score; (4) brain infarct size at 48 +/-24 hours; (5) EQ-5D-5 L score, and (6) WHODAS 2.0 score. Other outcomes are: the *primary safety outcome* serious adverse events; and the incremental cost-effectiveness, and cost utility ratios. The *analysis sets* include (1) the intention-to-treat population, and (2) the per protocol population. The sample size is estimated to 800 patients (5% type 1 and 20% type 2 errors). All analyses are adjusted for the protocol-specified stratification variables (nationality of centre), and the minimisation variables. In the analysis, we use ordinal regression (the primary outcome), logistic regression (binary outcomes), general linear model (continuous outcomes), and the Poisson or negative binomial model (rate outcomes).

**Discussion:**

Major adjustments compared with the original statistical analysis plan encompass: (1) adjustment of analyses by nationality; (2) power calculations for the secondary outcomes; (3) to address the multiplicity problem using of a fixed-sequence testing procedure starting with the primary outcome followed by the secondary outcomes ordered according to falling power; (4) assignment of worst possible score to patients who are not alive at the planned date of measurement of the continuous scores; (5) improved imputations; (6) outline of a supplementary exploratory analysis of the temperature measurements and time to death; and (7) substantial reduction of sample size.

**Trial registration:**

Clinicaltrials.gov, identifier: NCT01833312. 4 April 2013.

**Electronic supplementary material:**

The online version of this article (doi:10.1186/s13063-017-2302-z) contains supplementary material, which is available to authorized users.

## Introduction

The EuroHYP-1 trial is a multicentre, randomised, superiority phase III international clinical trial with masked outcome assessment testing the benefits and harms of therapeutic cooling in awake adult patients with acute ischaemic stroke (website: http://www.eurohyp1.eu). The trial is designed according to the SPIRIT guidelines, and the background, design, and rationale have previously been published [1]. The EuroHYP-1 trial protocol has been available online on www.ClinicalTrials.gov since the start of the trial on 4 April 2013. The trial is endorsed and supported by European Clinical Research Infrastructures Network (ECRIN) (www.ecrin.org).

Here we describe the updated detailed statistical analysis plan that has been finalised while data collection in the EuroHYP-1 trial is underway, and to which all data analyses in the main publication of the EuroHYP-1 trial results will adhere. A detailed statistical analysis plan was part of the protocol, and the Steering Group of the EuroHYP-1 trial unanimously approved the statistical analysis plan on 30 March 2016. The present amendments of the original statistical analysis plan focus on the primary and the secondary outcomes. The amendments have been made in order to make the analysis more concrete and transparent. Patient recruitment of 800 patients is expected to be completed, and the final follow-up is predicted to occur in 2018–19, after which the database will be locked and data will be analysed.

## Methods

### Objective of the EuroHYP-1 trial

The primary aim of the EuroHYP-1 trial is to determine whether systemic cooling to a target temperature of 34–35 °C, started within 6 hours of onset of stroke and maintained for up to 12 hours thereafter, improves outcomes in patients with acute ischaemic stroke. Inclusion and exclusion criteria are described in the design article in Tables 1 and 2 [1].

**Table 1:** Baseline characteristics of the participants in the EuroHYP-1 trial

Baseline characteristics	Intention-to-treat population		Per protocol population	
	Intervention group	Control group	Intervention group	Control group
Centre				
- name of centre 1, N (%)				
- name of centre 2, N (%)				
- name of centre 3, N (%)				
- etc				
Intention to perform thrombolysis, N (%)				
Surface cooling, N (%)				
Males, N (%)				
Stroke severity (NIHSS), mean (SD), N				
Age ≤ 65 years, N (%)				
Age, mean (SD), N				
Visible ischaemic lesion on brain imaging, N (%)				
Time since symptom onset, N ≤ 4 hours (%)				

*NIHSS* National Institutes of Health Stroke Score, *SD* standard deviation

**Table 2:** Power (based on an α = 0.05) of the secondary outcomes and serious adverse events provided inclusion of 800 patients into the EuroHYP-1 trial

Outcome	Proportion or mean value in control group	Standard deviation in the control group for continuous outcome	Minimal relevant intervention effect - absolute risk reduction relative to control group	Power
Score of NIHSS at 91 +/-14 days (sample size = 800)	8 points^a^	5 points^a^	2 points^a^	1.00
Serious adverse events at 91 +/-14 days (sample size = 800)	20%	NR	10%	0.98
Death or dependency, defined as modified Rankin score > 2 at 91 +/-14 days (sample size = 800)	63%	NR	7.25%	0.55
Brain infarct size at 48 +/-24 hours (sample size = 800)	10 ml^a^	15 ml^a^	2 ml^a^	0.47
EQ-5D-5 L score at 91 +/-14 days (sample size = 800)	0.50 points	0.40 points	0.05 points	0.42
Death at 91 +/-14 days (sample size = 800)	17%	NR	3.84%	0.33

*NIHSS* National Institutes of Health Stroke Score, *EQ-5D-5 L* EuroQoL quality-of-life scale, *NR* not relevant

^a^Assumed values

The null hypothesis is that there is no difference in death and disability as defined by a score on the modified Rankin Score (mRS) scale measured at 91 +/- 14 days after randomisation of the patient.

**Fig. 1:**
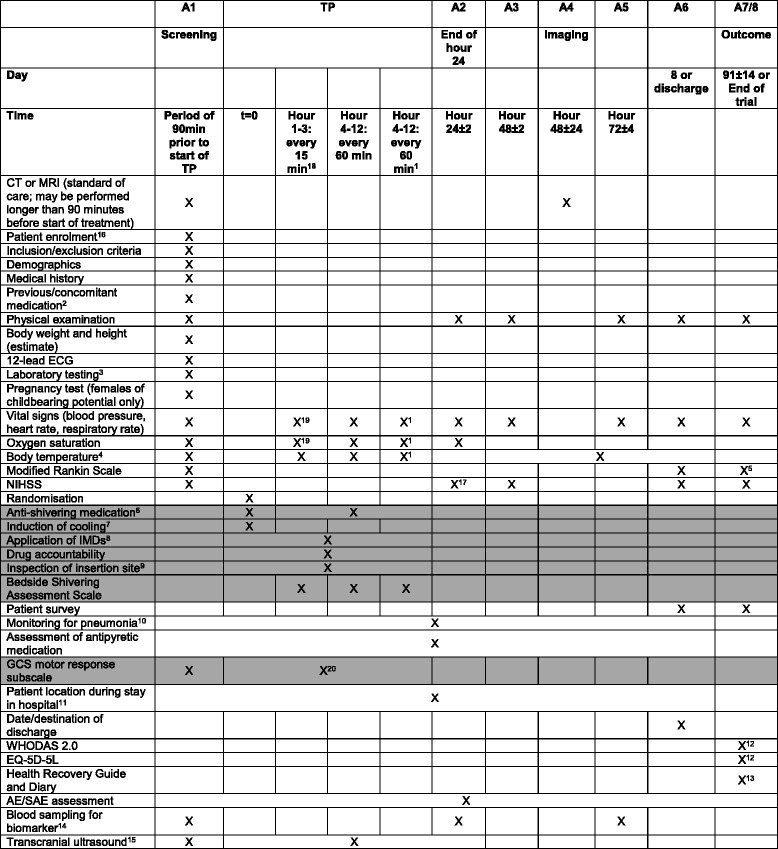
Overview of patient schedule and data collection in the EuroHYP-1 trial (*grey tone* only for the experimental group). 1 Re-warming: hypothermia group only. 2 Previous medication including alteplase. 3 Includes sodium, potassium, magnesium, creatinine, urea, gamma-glutamyl transpeptidase, ASAT, ALAT, alkaline phosphatase, blood glucose; haemoglobin, haematocrit, erythrocytes, leukocytes, platelets, INR. Further samples may be taken throughout the study at the discretion of the investigator. 4 Body temperature will be assessed according to local clinical practice with tympanic, bladder, or rectal temperature measurement, except in patients randomised to therapeutic hypothermia from start of treatment phase (TP, beginning of hour 1) until end of re-warming period, when bladder or rectal thermal probes will be used. During TP, body temperature will be assessed every 15 min during the first 3 hours (except at time points t = 0 min and t = 15 min) and every 60 min thereafter in patients randomised to therapeutic hypothermia, every 60 min (except at time point t = 0 min) in patients randomised to best medical treatment alone, subsequently in all patients at 8-hour intervals until A6 (day 8 or day of discharge from hospital, whichever occurs first). 5 The mRS assessment at outcome assessment (A7) will be recorded using a digital video camera. The clip will then be transferred to the EuroHYP-1 outcome adjudication web portal. 6 Anti-shivering medication: induction: buspirone 10 mg p.o./pethidine 50 mg i.v. (2 min); repeat doses of 10 mg buspirone p.o. may be administered as long as a maximum dose of 30 mg/24 h is respected; a bolus of pethidine 25 mg i.v. may be given as long as an interval of at least 30 min is respected and a maximum dose of 500 mg/24 h is not exceeded. 24 h-doses include induction bolus. For the prevention and treatment of opioid-induced nausea and vomiting, a 5HT3RA may be administered as support medication. 7 Induction of cooling: 20 ml/kg estimated bodyweight 4 °C isotone saline or Ringer´s lactate over a period of 30–60 min; EMCOOLS Brain.Pad, if available. 8 IMDs permitted for cooling: EMCOOLS Brain.Pad (for induction of cooling only); Medivance/Bard Arctic Sun temperature management system with heat exchange control unit Arctic Sun 2000 or Arctic Sun 5000 and ArcticGel Pads; MTRE CritiCool temperature management system with heat exchange control unit CritiCool, accessoires and CureWrap; Zoll IVTM system with heat exchange control unit CoolGard 3000 or Thermogard XP, CoolGard start-up kit and intravascular temperature management catheters ICYy 3893 AE or ICY 3893 CO. 9 If endovascular cooling is performed, the catheter insertion site must be visually inspected for detection of bleeding/haematoma in 3-hour intervals during TP and once 3 hours after removal of the intravascular catheter. 10 Monitoring for pneumonia includes monitoring of oxygen saturation and body temperature, physical examination (auscultation, percussion) and, if clinically indicated, chest X-ray. Monitoring for signs of pneumonia must be performed from screening assessment (A1, within 90 minutes before the start of the treatment phase TP) until A6 (day 8 or day of discharge from hospital, whichever occurs first). 11 Patient location during stay in hospital must be assessed at 12:00 hours on each day in hospital. 12 WHODAS 2.0 questionnaire and EQ-5D questionnaire must be filled in by the patient or his/her relative/carer at outcome assessment (A7). 13 Health Recovery Guide and Diary: Section 6: filled in by the patient every day from discharge to V7; Section 7: filled in by the carer/relative prior to V7. 14 For participation in the biomarker sub-study a special informed consent form must be filled in by the patient or his/her legal representative. Assessment at End of Hour 24 ± 2 hours. 15 Only selected study sites. 16 Informed consent will be obtained in accordance with national regulatory requirements. 17 NIHSS assessment at End of Hour 24 ± 2 hours. 18 Starting at t = 30 min. 19 Every 60 minutes only. 20 Prior to intended repeat administration of pethidine. *CT* computed tomography, *ECG* electrocardiogram, *EQ-5D-5 L* EuroQoL quality-of-life scale, GCS Glasgow coma scale, *MRI* magnetic resonance imaging, *NIHSS* National Institutes of Health Stroke Score, *SAE* serious adverse event, *WHODAS* World Health Organization Disability Assessment Schedule

For an overview of patient schedule and data collection see Fig. 1.

### The sample size

Originally, we planned to demonstrate or reject an absolute difference of 7% between the intervention groups (equivalent to an odds ratio (OR) of 0.74) and allowing for 3% loss to follow up in a sample size of 1500 randomised patients with a type 1 error risk of 5% and a type 2 error risk of 10% (EuroHYP-1, EudraCT number 2012-002944-25, version 2, 20 December 2012). Due to exceptionally slow enrolment, we realised that this target was no longer realistic. Accordingly, in 2014, the sample size was downgraded to 800 patients based on considerations explained in version 4 of the protocol (EuroHYP-1, EudraCT number 2012-002944-25, European Database on Medical Devices (EUDAMED) number CIV-12-09-008821, planned trial period July 2013 to March 2017 (45 months), version 4, date 29 June 2015). Currently, the project has been prolonged until 31 July 2018.

### Adjusting variables

The randomisation is stratified according to the nationality of the participating centres and, within each stratum, the patient allocation is based on probabilistic minimisation (80:20) using the following factors:

- Intention to perform thrombolysis (yes compared to no).
- Method of cooling (surface compared to endovascular).
- Sex (male compared to female).
- Stroke severity (National Institutes of Health Stroke Score (NIHSS)) 6–12 compared to 13 or higher).
- Age (≤ 65 years compared to > 65 years).
- Visibility of a relevant ischaemic lesion on the first brain imaging (yes compared to no).
- Time since symptom onset (≤ 4 hours compared to 4–6 hours).

The primary analyses will be adjusted by the protocol-specified stratification variable country and the seven minimisation factors.

### Flow of patients and baseline characteristics

**Fig. 2:**
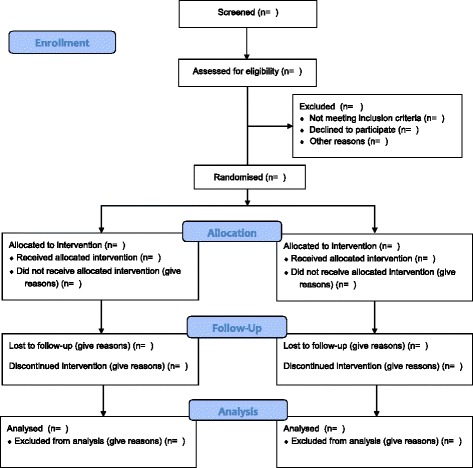
Flow of participants in the EuroHYP-1 trial

The flow of patients will be reported according to the CONSORT guidelines (Fig. 2). Patients’ baseline characteristics will be reported in a table (Table 1) for the intention-to-treat population and for the per protocol population.

### Definition of the efficacy and safety outcomes

Outcomes in the original statistical analysis plan (SAP) included in the EuroHYP-1 protocol are defined as primary, secondary, and exploratory. In the present SAP, we deal only with the primary and secondary outcomes as well as one safety outcome and one health-economic outcome. For information on the exploratory outcomes mentioned and defined in the EuroHYP-1 trial protocol and the analyses of these outcomes the reader is therefore referred to the protocol [1].

#### The primary outcome

The primary outcome is degree of disability post stroke as measured by the seven-level modified Rankin Scale measured in the period 91 ± 14 days after the patient’s randomisation.

#### The secondary outcomes originally selected

The order of secondary outcomes as originally selected in the protocol was:

- Death or dependency, defined as a score on the mRS > 2 at outcome assessment in the period day 91 ± 14 days after randomisation. Type: binary.
- Death at outcome assessment in the period day 91 ± 14 days after randomisation. Type: binary.
- Score on National Institute of Health Stroke Scale (NIHSS) at outcome assessment in the period day 91 ± 14 days after randomisation. Type: continuous.
- EuroQoL quality-of-life (EQ-5D-5 L) score at outcome assessment in the period day 91 ± 14 days after randomisation. Type: continuous.
- World Health Organization Disability Assessment Schedule II (WHODAS 2.0) score at outcome assessment in the period day 91 ± 14 days after randomisation. Type: continuous.
- Brain infarct size at imaging assessment in the period 48 ± 24 hours after randomisation. Type: continuous.

#### Safety outcomes

- Proportion of participants with at least one serious adverse event (SAE) until outcome assessment in the period day 91 ± 14 days after randomisation. Type: binary.
- Number of SAEs per patient until outcome assessment in the period day 91 ± 14 days after randomisation. Type: rate.

#### Health economic outcome

- Incremental cost utility (cost per quality-adjusted life years (QALY)) and incremental cost effectiveness (cost per death or disability averted) ratios. Type: continuous.

### The analysis sets

The analysis populations for the statistical analyses of this trial include the intention-to-treat population, the per protocol set of patients, and the ‘learning curve’ population.

#### The intention-to-treat population of patients

The intention-to-treat (ITT) population is defined as all randomised patients classified according to the intervention to which they were randomised (experimental and control intervention groups) [2].

#### The per protocol set of patients

The per protocol set of patients is the subset of all randomised patients without major protocol violations and classified according to the intervention to which they were randomised. Major protocol deviations encompass: (1) patients in the intervention group who do not reach a body temperature of ≤ 35.0 °C within 3 hours after induction of cooling; or (2) patients randomised in the intervention group who do not achieve at least 6 hours of body temperature at a level of ≤ 35.0 °C during the period from beginning of hour 1 to the end of hour 12 of cooling. According to this definition, the per protocol population will be coded and defined in the data set before data lock, so it cannot undergo changes subsequently.

#### The ‘learning curve’ population

The EuroHYP-1 trial is a very large and complex trial with multiple nations and sites investigating a complex intervention. To account for this, we define a ‘learning curve’ analysis set, where we will analyse independently the first five participants included at each centre and compare those results to the results of all participants minus the first five.

### Statistical analyses

#### Analytical principles

Analyses will be conducted according to the intention-to-treat principle [2] if not otherwise stated.

All primary tests of significance will be two-sided with alpha = 5%. Analyses will be conducted blinded with the two intervention groups coded as, e.g. 0 and 1. Two conclusions will be drawn, one assuming 0 is the intervention group and 1 is the control, and one assuming the opposite. After two abstracts with two conclusions have been written and both accepted by the Steering Group, the code will be broken.

The primary analyses of primary, secondary, and exploratory outcomes will be those of the intention-to-treat population adjusted for the protocol-specified stratification variable and the minimisation variables, and if necessary using data sets generated using multiple imputation.

#### Missing values of the primary outcome

By definition, none of the adjusting variables will have missing values (otherwise the stratification and minimisation could not have been conducted). So only the outcome value may be missing. Furthermore, we assume there are no useable auxiliary variable, i.e. no other variable that has a moderate to high correlation with the outcome (|r| > 0.40) and yet is not intended to be a predictor in the regression model. In this situation, the problem is easily solved. If we are willing to assume that the data are missing at random, the maximum likelihood analysis reduces to a complete case analysis [3, 4]. Consequently, we simply delete participants that are missing on the outcome and estimate the regression with the remaining participants. However, if useable auxiliary variables have been identified, a multiple monotone imputation will be conducted, which will include the primary outcome and the covariates of the analytical model plus the auxiliary variables. As a sensitivity analysis to assess the potential impact of outcome values missing not at random (MNAR), we will conduct a worst/best case scenario imputation, i.e. a single value imputation of the missing values. The imputations use a minimum value or a maximum value (0 respectively 5 of the modified Rankin scale in that we assume that a patient who died will not have the mRS value missing). Missing values are imputed by the minimum value in one intervention group, and in the other intervention group by the maximum value and vice versa. Once the randomisation code is broken the results of the two comparisons may be interpreted.

#### Power of the original secondary outcomes

As the sample size is based on the primary outcome, we have calculated the power of each of the originally planned secondary outcomes given the sample size of 800 patients.

Death day 91 ± 14 days after randomisation: the implied clinically relevant minimal change in mortality that needs to be detected may be calculated based on (1) the mortality of 17% found in the meta-analysis quoted in the protocol [1]; (2) the assumption that wherever the mRS is dichotomised, the odds ratio for treatment versus control will be the same, the ‘common odds ratio’; and (3) the requirement that this common odds ratio should not be larger than 0.74 (see the sample size calculation). Let the unknown change in mortality in the experimental group be x. If we assume the OR for the dichotomy mRS > 5 compared to mRS ≤ 5 will be 0.74 and the mortality of the control group is 17% as quoted in the meta-analysis, we may find x by solving the equation OR = ((0.17–x)/(0.83 + x))/(0.17/0.83) = 0.74 to obtain x = 0.0384 giving a mortality of 0.1700 – 0.0384 = 0.1316 in the cooled patients versus 0.1700 in the controls. Using a sample size of 800 participants, the power is found to be 0.33.

Death or dependency (mRS > 2) 91 ± 14 days after randomisation: using the same reasoning, the power of this outcome can be calculated by solving the equation (0.63–x)/(0.37 + x))/0.63/0.37 = (0.63 – x)0.63/(0.37 + x)0.37 = 0.74 as 63% of the patients in the meta-analysis had mRS > 2 implying x = 0.0725. The power may then be calculated to be 0.55.

NIHSS score 91 ± 14 days after randomisation: assuming a minimal relevant difference of 2 points, a standard deviation (SD) of 5 points [5], an α of 0.05, and a sample size of 800 participants, the power may then be calculated to 1.00.

EQ-5D-5 L score 91 ± 14 days after randomisation: assuming a minimal relevant difference of 0.05 points, a SD of 0.40 points [6–9], an α of 0.05, and a sample size of 800 participants, the power may then be calculated to 0.42.

WHODAS 2.0 score 91 ± 14 days after randomisation: we originally planned to analyse the World Health Organization Disability Assessment Schedule II (WHODAS 2.0) score at outcome assessment in the period day 91 ± 14 days after randomisation. However, due to difficulties in assessing the clinical meaning, we are not able to calculate the power, and we will thus analyse this as an exploratory outcome.

Brain infarct size 48 ± 24 hours after randomisation: assuming a minimal relevant difference of 2 ml, a SD of 15 ml [10], an α of 0.05, and a sample size of 250 participants, the power may then be calculated to 0.18. Accordingly, this outcome will be exploratory.

Number of patients with at least one serious adverse events 91 ± 14 days after randomisation: assuming a control group event proportion of 20% of patients with at least one SAE, an intervention group event proportion of 10% [11], an α of 0.05, and a sample size of 800 participants, the power may then be calculated to 0.98.

Mean number of SAEs per patients: As we do not have prior data to indicate a probable minimal relevant difference, we cannot calculate power for this outcome. These data will be analysed as an exploratory outcome.

The results of calculations of the power are all shown in Table 2. These results are used to determine the sequence in which the secondary outcomes should be tested using the fixed-sequence approach, i.e. the sequence will be as follows:

1. Score on NIHSS at outcome assessment in the period day 91 ± 14 days after randomisation.
2. Proportion of participants with at least one serious adverse event (SAE) until outcome assessment in the period day 91 ± 14 days after randomisation.
3. Death or dependency, defined as a score on the mRS > 2 at outcome assessment in the period day 91 ± 14 days after randomisation.
4. Brain infarct size at imaging assessment in the period 48 ± 24 hours after randomisation.
5. EQ-5D-5 L score at outcome assessment in the period day 91 ± 14 days after randomisation.
6. Death at outcome assessment in the period day 91 ± 14 days after randomisation.

The exploratory outcomes are:

1. WHODAS 2.0 score at outcome assessment in the period day 91 ± 14 days after randomisation.
2. Number of SAEs per patient until outcome assessment in the period day 91 ± 14 days after randomisation.

### Presentation of results in tables

Tables 3, 4, 5 and 6 show how the results of the analysis of the primary outcome (Table 3), the binary secondary outcomes (Table 4), the continuous secondary outcome and exploratory outcomes (Table 5), and the SAE data (Table 6) will be presented.

**Table 3:** Comparison of the distributions of the modified Rankin scale (mRS) between the two intervention groups in each analysis set (intention-to-treat or per protocol)

Population studied	Intervention group	mRS = 0	mRS = 1	mRS = 2	mRS = 3	mRS = 4	mRS = 5	mRS = 6	Common OR	P of difference
		N (%)	N (%)	N (%)	N (%)	N (%)	N (%)	N (%)	(95% CI)	(reference = group 0)
Intention-to-treat (ITT)	Group 0									
	Group 1									
Per protocol (PP)	Group 0									
	Group 1

**Table 4:** Comparison of the distributions of the secondary binary outcomes between the two intervention groups in each analysis set (intention-to-treat and per protocol)

Population	Intervention group	Outcomes					
		Death			mRS > 2		
		N (%)	Relative risk (RR) (95% CI)	P	N (%)	RR (95% CI)	P
Intention-to-treat (ITT)	Group 0						
	Group 1						
Per protocol (PP)	Group 0						
	Group 1						

*mRS* modified Rankin score, *CI* confidence interval

**Table 5:** Comparison of distributions of the secondary continuous outcomes between the two intervention groups in each analysis set (intention-to-treat or per protocol)

Outcome	Population	Intervention group	Percentiles			N	Mean (SD)	(Minimum, maximum)	*P* of difference
			25%	50%	75%				
NIHSS score Range of possible scores at 91+/-14 days (RS): 0 to 42 Worst = 42	Intention-to-treat (ITT)	Group 0							
		Group 1							
	Per protocol (PP)	Group 0							
		Group 1							
EQ-5D-5 L score at 91+/-14 days RS: 0 to 100 Worst = 0	ITT	Group 0							
		Group 1							
	PP	Group 0							
		Group 1							
Brain infarct size at 48 +/-24 hours Worst = 1	ITT	Group 0							
		Group 1							
	PP	Group 0							
		Group 1							
WHODAS 2.0 score at 91+/-14 days	ITT	Group 0							
		Group 1							
	PP	Group 0							
		Group 1							

*SD* standard deviation, *NIHSS* National Institutes of Health Stroke Score, *EQ-5D-5 L* EuroQoL quality-of-life scale

**Table 6:** Amendments made relative to the original statistical analysis plan published in the protocol

Topic described in original statistical analysis plan	Handling of topic in the present amended statistical analysis plan	Reason for action
No adjustment for nationality.	Adjustment for nationality	To improve the power by preventing upward bias of the standard error of the outcome.
Power calculation of secondary outcomes is missing	Calculation of power conditional on sample size	To be used when defining the test ordering of the secondary outcomes
Difficulties involved in interpreting an effect on a secondary outcome that can only be measured in surviving patients was not addressed	The worst possible score is assigned to the dead patients	A surplus of patients in one group relative to the other group may die before the outcome is measured. In the other group the corresponding surviving patients may (or may not) have very poor outcomes
No multiplicity adjustment	The fixed-sequence testing procedure will be applied with the primary outcome to be tested first and followed by the secondary outcomes ordered according to falling power	To keep the family-wise error rate ≤ 0.05
A search for auxiliary variables and if found followed by imputation of the primary outcome was not considered	Missing value handling procedure revised accordingly	To improve the efficiency of a multiple imputation of missing values of the primary outcome
The analytic potential of the exploratory temperature data was not expanded on	An outline of an exploratory mixed model analyses of the temperature data and Cox analyses of time to death with censoring at end of treatment and at 91 days +/- 14 days is now included	To assess if the temperature has an impact on short-term and long-term mortality and supplement the result of the analysis of the second secondary outcome
Sample size of 1500 participants	Sample size reduced to 800 participants	Due to exceptionally slow enrolment, we realised that this target was no longer realistic

#### Multiplicity

We cannot claim a significant beneficial effect of the intervention in the trial if the primary outcome is neutral and a secondary outcome is statistically significant. Therefore, any testing addressing the multiplicity problem must not reduce the power of the test of the primary outcome as compared to the situation where we declare the secondary outcomes exploratory and only test the primary outcome using α = 0.05. Consequently, the primary outcome must be tested initially and using α = 0.05. These requirements may be fulfilled if we use a pre-specified testing sequence (fixed-sequence procedure) where each outcome is tested at α = 0.05 [12]. However, as soon as a non-significant result is obtained, the testing is stopped. However, for exploratory purposes we will calculate the *P* values of the remaining tests and present them. We will use the fixed-sequence procedure starting with the primary outcome and followed by the secondary outcomes, and the exploratory outcomes ordered according to descending power (see Table 2).

#### Subgroup analysis

The duration of the cooling intervention was reduced from 24 hours to 12 hours after the recruitment of 50 participants. To investigate this protocol change, we will conduct a subgroup analysis for the primary and secondary outcomes. In the analysis, we will compare the participants who were included before the protocol change to participants included after the protocol change.

#### Sensitivity analyses

The analyses of the primary and secondary outcomes will be repeated using the per protocol population and the ‘learning curve’ population.

### Analysis of the primary and the secondary outcome

#### The primary outcome

The primary efficacy variable, the score on the mRS at the outcome assessment (day 91 ± 14 days), will be determined with ordinal (with more than two categories) logistic regression. The assumption about proportional odds will be accepted if the difference between the frequency in group 1 and that of group 2 in the various categories are all positive and different from 0 or are all negative and different from 0. If the assumption of the ordinal regression analysis model is not fulfilled, the groups will be compared using a non-parametric method (van Elteren and stratification by nationality of centre) and the result will be the primary result. If the assumption of the model is fulfilled, the result of the adjusted analysis will be the primary result.

#### The secondary and the exploratory outcomes

Frequencies and percentages per group as well as risk ratios with 95% confidence interval (CI) will be reported for binary outcomes. Continuous variables and rate variables will be summarised using mean, standard deviation, 25, 50 and 75 percentiles, and minimum and maximum values.

Logistic regression for binary quantities, the general linear univariate model for continuous outcomes, and the Poisson distribution or negative binomial distribution for rate outcomes will be used. If the assumptions of the Poisson or negative binomial models are not fulfilled with reasonable approximation, a non-parametric method (van Elteren adjusted by nationality of centre) will be used.

#### Outline of exploratory analysis of the temperature variables and time to death

Assuming that the temperature is measured at the same times relative to the time of the start of intervention, a mixed model may be used to characterise the time course of those patients who do not die during the treatment. The model is given by temperature = INT time time*INT, where INT is the intervention indicator. Additionally, the time to death from start of treatment with censoring at end of treatment and at 91 days +/-14 days including baseline variables measured at or prior to start of treatment may be analysed using a Cox proportional hazards model. The mean values of all the actual measured temperatures in the intervention group will be displayed in a graph with mean +/- 2 standard errors.

#### Health economic evaluation

The economic assessments will be conducted in alignment with the Consolidated Health Economic Evaluation Reporting Standards (CHEERS) statement [13]. The prospective analysis will determine the cost per QALY gained with systemic cooling compared with standard care over a 3-month period. Acute hospital post-discharge resources will be considered in the evaluation. Procedural costs for systemic cooling will be obtained with a bottom-up micro-costing approach in order to identify all relevant cost components of the procedure and value each component for all individual patients, using procedure duration, staff, and number of medical devices as variables. All-cause hospital admissions within 3 months of the first treatment will also be included in cost computations. Non-hospital resources are not considered in view of the short follow-up duration.

Cost of systemic cooling will be the manufacturer’s price, and staff costs will be estimated from gross salaries. Hospital inpatient costs will be estimated using current average national cost of each patient’s diagnosis-related group (DRG), adjusted for actual length of stay, and resources consumed during hospitalisation. Hospital re-admissions will be based on tariffs. All costs will be in Euros (€) and are not discounted. Health-related quality of life collected using the EQ-5D-5 L self-administered questionnaire at baseline and 3 months will be used to elicit utility values based on country-specific tariffs. The difference in QALYs will be estimated as the difference in the area between the utility curves for the two groups.

We will undertake a fully pooled analysis for clinical outcomes and a country-specific analysis for costs (based on the countries with the highest patient volume) [14].

The cost-utility analysis will estimate incremental costs per QALY and the cost-effectiveness incremental cost per death or disability (as defined in the secondary outcomes) averted. Between-group comparisons of QALYs and costs will be performed with the statistical test appropriate for their distribution, with a significance threshold of 0.05. A joint comparison of costs and effects will be performed by nonparametric bootstrapping with 1000 resamples and the result of the bootstrap replications presented on the cost-effectiveness plane (scatterplot).

### Overview of changes to the original statistical analysis plan for the primary and the secondary outcomes

Table 7 presents an overview of the changes relative to the original statistical analysis plan and explain the reasoning behind. The only substantial changes relative to the original plan includes that we now: (1) adjust the analyses by the protocol-specified stratification variable, i.e. nationality to improve the power [15]; (2) power calculations for secondary outcomes; (3) address the multiplicity problem by using a fixed-sequence procedure starting with the primary outcome followed by the secondary outcomes ordered according to falling power; (4) due to the low power involved in detecting small (1–5%) differences in mortality between the groups, assign worst possible score to patients who are not alive at the planned date of measurement of the continuous scores; (5) include auxiliary variables in the potential multiple imputation of missing value of the primary outcome to improve the efficiency of the imputation; and (6) outline a supplementary exploratory analysis of the temperature measurements and time to death; (7) substantial reduction of sample size.

**Table 7:** Total number of serious adverse events (SAEs), and number of patients with at least one SAE groups in each analysis set (intention-to-treat or per protocol)

Types of events	Group 0			Group 1		
	Events, N	Patients with at least one event, N	Patients assessed, N	Events, N	Patients with at least one event, N	Patients assessed, N
Any event						
Event type 1						
Event type 2						
Event type 3						
Etc						

## Discussion

With this updated detailed statistical analysis plan we present the different analyses in the main publication of the EuroHYP-1 trial to avoid risks of outcome reporting bias and data driven results [16, 17]. Of the pre-specified outcomes in the trial, we choose to report only the primary outcome, the secondary outcome, two exploratory outcomes including serious adverse events in the main publication because of the complexity of the remaining safety variables and the detailed economical quantities that require separate publications.

### The interpretation of the effect on outcomes which may only be measured in surviving patients

Formally, three of the original secondary outcomes (NIHSS, EQ-5D-5 L, and infarct size) are only measurable in patients who survive until these outcomes were planned to be measured. This may make the interpretation of a significant effect on each of the last four secondary outcomes somewhat difficult when the power of detecting a small difference between the mortalities in the two groups is low. A surplus of patients in one intervention group relative to the other group may die before the outcome is measured. In the other intervention group, the corresponding surviving patients may (or may not) have very poor outcome values which may make the first group look good if the mortalities do not differ significantly.

Given the sample size of 800 patients and assuming the mortality in one group is 0.17 (e.g. in the control group, see above) the probability of not detecting an absolute reduction in mortality in the experimental group of 0.01, 0.02, 0.03, 0.04, and 0.05 is 0.93, 0.89, 0.81, 0.70, and 0.57, respectively. Thus, differences in mortality of the intervention groups of 5% or less are likely to remain undetected with a relatively high probability. Therefore, we have elected to include the dead patients in the comparison between the two intervention groups by assigning the worst possible score to the dead patients. The EQ5D5L is used to estimate QALYs gained in each arm of the trial population. QALYs are estimated by summing up the time spent in each state multiplied by the quality of life attributed to this state. By definition, the value of QALYs is zero from the time of death onwards. Total QALYs will be computed and reported for each patient for the entire duration of the follow-up.

For the study of prognostic markers in the EuroHYP-1 trial, we will apply similar principles as in the Clarithromycin in Patients with Stable Coronary Heart Disease trial (CLARICOR) trial [18]. The study of prognostic markers will not be part of the primary publication.

In conclusion, major adjustments compared with the original statistical analysis plan encompass: (1) adjustment of analyses by nationality; (2) power calculations for the secondary outcomes; (3) use of a fixed-sequence testing procedure starting with the primary outcome followed by the secondary outcomes ordered according to falling power to address the multiplicity problem; (4) assignment of worst possible score to patients who are not alive at the planned date of measurement of the continuous scores; (5) improved imputations; (6) outline of a supplementary exploratory analysis of the temperature measurements and time to death; and (7) substantial reduction of sample size.

## Additional file

Additional file 1: List of principal investigators in EuroHYP-1 and sites. (DOCX 32 kb)

## Abbreviations

CHEERS: Consolidated health economic evaluation reporting standards
CLARICOR: Clarithromycin in Patients with Stable Coronary Heart Disease trial
DRG: Diagnosis-related group
EQ-5D-5 L: EuroQoL quality-of-life scale
EUDAMED: European Database on Medical Devices
EudraCT: European Clinical Trials Database
ITT: Intention-to-treat
MNAR: Missing not at random
mRS: Modified Rankin score
NIHSS: National Institutes of Health Stroke Score
OR: Odds ratio
QALY: Quality-adjusted life years
SAE: Serious adverse event
SAP: Statistical analysis plan
SD: Standard deviation
WHODAS: World Health Organization Disability Assessment Schedule
