# Supplementary material for: Statistical analysis plan for the EuroHYP-1 trial: European multicentre, randomised, phase III clinical trial of the therapeutic hypothermia plus best medical treatment versus best medical treatment alone for acute ischaemic stroke
Source: Trials. 2017 Nov 29;18:573. doi: 10.1186/s13063-017-2302-z (PMC5706304; doi:10.1186/s13063-017-2302-z)
Supplement: Additional file 1: — List of principal investigators in EuroHYP-1 and sites. (DOCX 32 kb) [file 13063_2017_2302_MOESM1_ESM.docx]

**Additional file 1**

List of principal investigators in EuroHYP-1 and sites (in alphabetical order):

Juan Francisco Arenillas-Lara, Hospital Clinico Universitario de Valladolid, Spain; Rajaram Bathula, Northwick Park Hospital, UK; Jörg Berrouschot, Klinik für Neurologie, Klinikum Altenburger Land, Altenburg, Germany; Hanne Krarup Christensen, Bispebjerg Hospital, Denmark; Charlotte Cordonnier, CHRU Lille, France; Ben Creagh-Brown, The Royal London Hospital, Barts Health NHS trust, UK; Anna Członkowska, Warsaw Institute of Psychiatry and Neurology, Poland; Philippe Desfontaines, Centre Hospitalier Chrétien, Liège Belgium; Christian Dohmen, Klinik und Poliklinik für Neurologie, Köln, Germanry; Rainer Dziewas, University Hospital Münster, Germany; Martin Griebe, Neurologische Universitätsklinik, Mannheim, Germany; Albrecht Günther, Universitätsklinikum Jena, Germany; Dalius Jatuzis, Vilnius University Hospital Santariskiu kliniko, Lithuania; Bernd Kallmünzer, Universitätsklinik Erlangen, Germany; Ralf Lindert, Royal Hallamshire Hospital, UK; Matthias Lorenz, Zentrum der Neurologie, Universität Frankfurt, Germany; Andreas Meisel, Center for Stroke Research Berlin, Germany; Dominik Michalski, Klinik und Poliklinik für Neurologie, Leipzig, Germany; Wolf-Dirk Niesen, Universitätsklinikum Freiburg, Germany; Francesco Orzi, Roma Azienda Ospedaliera Sant`Andrea, Italy; Kath Pasco, Royal Surrey County Hospital, UK; Richard Perry, Royal Liverpool University Hospital, UK; Bartlomiej Piechowski-Jozwiak, King’s College Hospital, London, UK; Sven Poli, Zentrum für Neurologie, Herti-Institut für klinische Hirnforschung, Tubingen, Germany; Peter Ringleb, Universität Heidelberg, Germany; Risto Roine, Turku University Hospital, Finland; Marta Rubiera, Hospital Vall D Hebron Barcelona, Spain; Ingo Schirotzek, Neurologische Klinik, Universitätsklinik Gießen und Marburger GmbH, Germany; Hauke Schneider, Universitätsklinikum Carl Gustav Carus, an der Technischen Universität Dresden, Germany; Joaquín Serena, Hospital Universitario de Girona Dr Josep Trueta, Spain; Igor Sibon, CHU Bordeaux / Hôpital Pellegrin, France; Nikola Sprigg, Nottingham University Hospital, UK; Alan Sweenie, Newcastle Royal Victoria Infirmary, UK; Kari Saastamoninen, University College London Hospital, UK; Götz Thomalla, University Medical Center Hamburg-Eppendorf, Germany; Serge Timsit, CHRU La Cavale Blanche, Brest, France; Geert Vanhooren, AZ Sint Jan Brugge-Oostende AV, Belgium; Marius Venter, Imperial College Healthcare NHS Trust, UK; and Katja Wartenberg, Klinik für Neurologie, Martin-Luther-Universität Halle-Wittenberg, Halle, Germany.
